# Olmesartan-Induced Ischemic Enteritis Complicated With Bowel Perforation: A Case Report and Literature Review

**DOI:** 10.7759/cureus.36660

**Published:** 2023-03-25

**Authors:** Dheeraj Alexander, Basel Abdelazeem, Mustafa Alnounou

**Affiliations:** 1 Internal Medicine, McLaren Health Care, Flint/Michigan State University, Flint, USA; 2 Gastroenterology, McLaren Health Care, Flint/Michigan State University, Flint, USA

**Keywords:** olmesartan-induced enteropathy, angiotensin receptor blockers, bowel perforation, ischemic enteritis, olmesartan

## Abstract

Olmesartan is a relatively new angiotensin receptor blocker used widely to control hypertension. Cases have been reported previously of enteropathy induced by olmesartan. Here, the authors report a case of olmesartan-induced ischemic enteritis complicated by bowel perforation. A 52-year-old male patient, during the treatment with olmesartan, developed severe abdominal pain of five-day duration. He underwent exploratory laparotomy for bowel perforation and surgical resection of the ischemic bowel segment. On a two-month follow-up after the discontinuation of olmesartan and the emergency surgery, the patient was symptom-free and functioning well. This rare report focuses on ischemic enteritis associated with olmesartan, describes the symptoms, and records the progression of this side effect and the corresponding treatment. Our case aims to raise awareness amongst physicians about the possibility of this severe complication and to point out that more research is still needed on its pathophysiology to better understand this drug.

## Introduction

Olmesartan is an angiotensin receptor-blocking (ARB) agent used in the management of hypertension. It can be used as a single agent to treat hypertension in the absence of comorbidities like cerebrovascular events, chronic kidney disease, heart failure, diabetes, and ischemic heart disease. Common side effects include headache (7%), dizziness (3%), upper respiratory infections (5%), hyperglycemia (>1%), and hypertriglyceridemia (>1%) [1]. Olmesartan-induced enteropathy has been increasingly reported in the literature since 2012; the most commonly reported histologies are villous atrophy and intraepithelial lymphocytosis [2]. In most of the previously reported cases, the discontinuation of olmesartan improved patient presentations [2-9].

We report a case of olmesartan-induced ischemic enteritis, which ultimately resulted in bowel perforation. The purpose of reporting this rare case is to raise awareness amongst physicians about the possibility of this severe complication and thereby educate their patients to seek immediate medical attention if the associated symptoms persist while on this medication.

## Case presentation

A Caucasian male in his 50s was admitted to our hospital with severe intermittent abdominal pain, nausea, and vomiting of five-day duration. For about two years, he had been taking olmesartan, an angiotensin II receptor blocker, to treat his hypertension. He was found to have a mild elevation in triglycerides six months ago, which was being managed with lifestyle modification. He denied taking any other medications or herbal remedies at home. The pain was not related to food intake. He has no other comorbidities. There is no history of past abdominal surgeries. The patient occasionally smokes; there is no prior history of alcohol or illicit drug use. On examination, there was minimal tenderness in the epigastric area. The laboratory results are summarized in Table 1. The patient was tachycardic at 110 beats per minute but normotensive at 128/85 mmHg. The patient was treated for sepsis and resuscitated with 2 L of normal saline. Pan-cultures were sent. An empiric parenteral antibiotic, intravenous piperacillin-tazobactam 3.375 g every eight hours, was initiated. Since admission, his abdomen has become more distended, and he has had intractable nausea and vomiting. The nasogastric tube was placed and connected to suction, and 500 mL of coffee ground content was removed from his stomach.

**Table 1:** Laboratory workup results at admission and on the day of discharge. WBC: white blood cells; Hb: hemoglobin level; BUN: blood urea nitrogen; S.Cr: serum creatinine; /µL: per microliter; g/dL: gram per deciliter; nmol/L: nanomoles per liter; mg/dL: milligrams per deciliter.

Laboratory test	Initial laboratory values (hospital day 1)	Discharge laboratory values (hospital day 21)	Normal range
WBC	26.13	12.13	4.5–11 × 10*3/µL
Hb	13.9	7.7	13.5–17.7 g/dL
Anion gap	16.5	12.4	4–12 nmol/L
BUN	34	24.3	9–27 mg/dL
S. Cr	1.4	2.1	0.6–1.5 mg/dL

**Figure 1:**
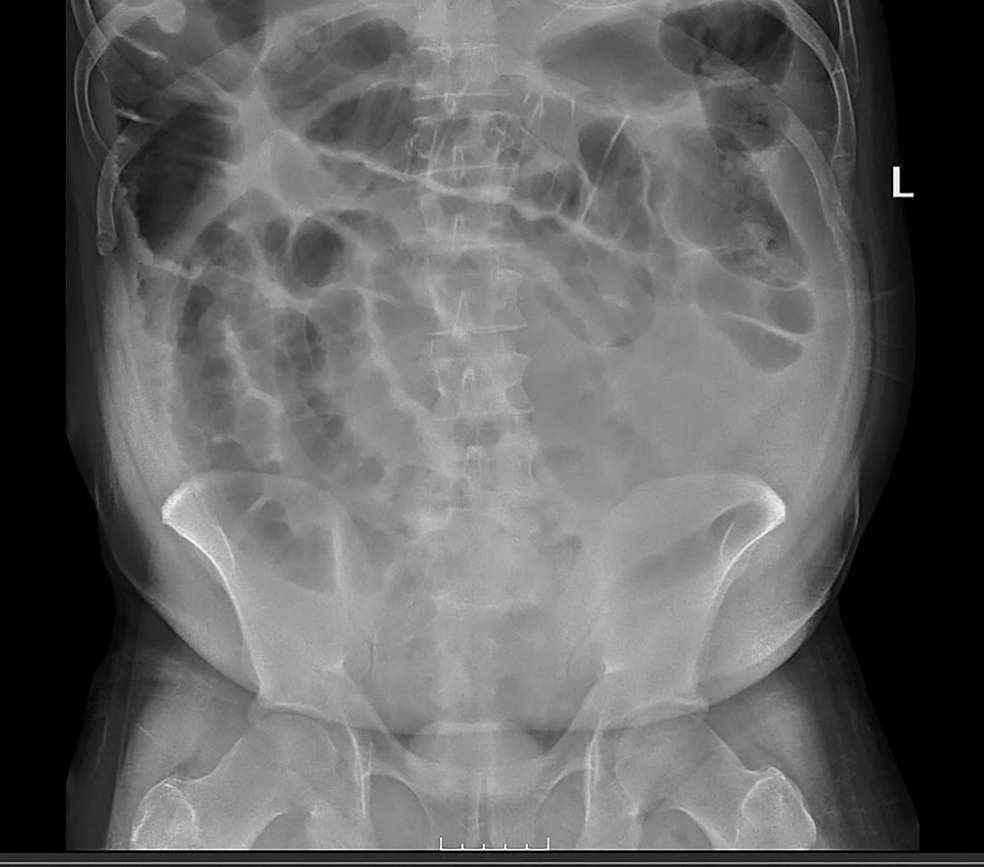
Abdominal X-ray showed diffuse ileus.

**Figure 2:**
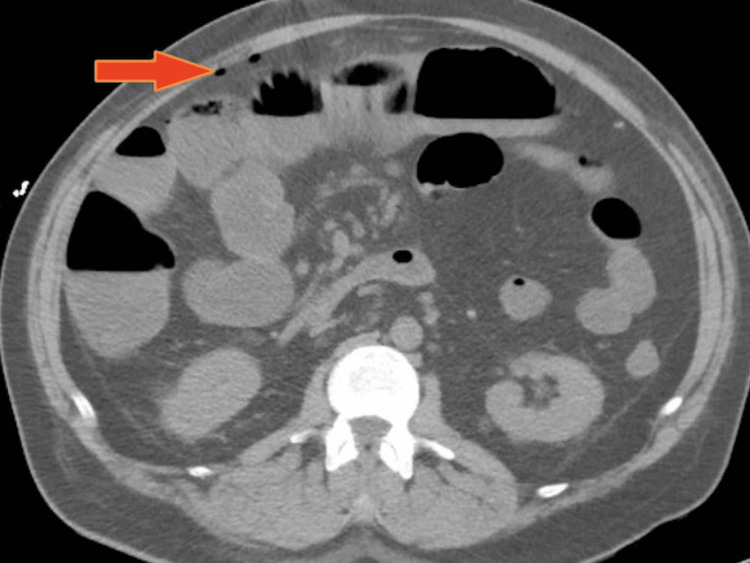
CT abdomen showing extra-intestinal foci of air suggestive of bowel perforation. The red arrow shows extra-intestinal foci of air.

An abdominal X-ray showed findings suggestive of ileus with no evidence of bowel obstruction or perforation (Figure 1). A computerized tomography (CT) angiogram of the abdomen and pelvis was negative for mesenteric ischemia or bowel obstruction (Figure 2). The small bowel follow-through series were also consistent with diffuse ileus and no frank obstruction. He underwent an upper endoscopy on day 2, which showed distal gastritis. As drug-related enteropathy and secondary ileus were suspected, olmesartan was discontinued on day 2. Due to worsening abdominal pain, a repeat CT abdomen was performed on day 6 of admission, which revealed bowel perforation.

**Figure 3:**
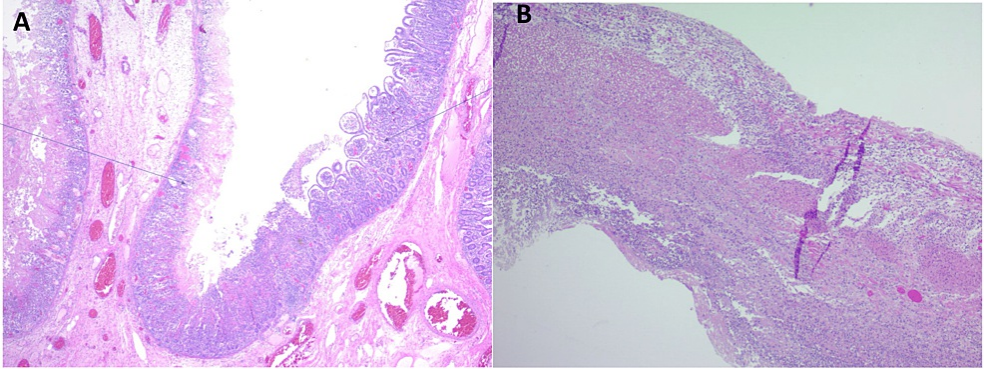
Histopathology of a segment of the intestine. (A) Showing an intact area of the small intestinal mucosa. Arrow shows the intact area of the small intestinal mucosa (B) showing necrosis involving all layers of the intestinal wall (transmural necrosis).

The patient underwent an emergent exploratory laparotomy, which revealed 25 to 30 cm of ischemia in the jejunal loop with a perforation. The ischemic segment of the small bowel was resected, and open abdomen negative pressure therapy (ABthera™) was placed in anticipation of a second look exploratory laparotomy. The patient was taken for a second look for exploratory laparotomy the next day, during which bowel anastomosis and abdominal closure were performed. Surgical pathology showed transmural ischemic changes in the resected segment of the small bowel (Figure 3). He was managed in the intensive care unit postoperatively for a total of 11 days. The patient’s clinical condition improved on postoperative day 3. Hemoglobin remained stable at around 7.7 g/dl, and the patient was hemodynamically stable. At the time of discharge, the patient was started on nifedipine for blood pressure management. In two months of follow-up, the patient was completely asymptomatic and blood pressure was well controlled on nifedipine.

## Discussion

Olmesartan is an ARB commonly used in managing hypertension and was approved for marketing in the United States in 2002 [1]. Olmesartan-induced enteropathy has been in the literature since 2012, when Rubio-Tapia et al. first reported a case series of 22 patients [3]. The most commonly reported histopathologies were villous atrophy and intraepithelial lymphocytosis. Olmesartan-induced enteropathy occurs after an average drug exposure duration of 3.1 years [3]. Table 2 illustrated a literature review of the reported cases and showed common presentations: chronic diarrhea, nausea, vomiting, abdominal pain, and weight loss. There has been complete recovery after the withdrawal of olmesartan in nearly all reported cases of this medication-induced enteropathy.

**Table 2:** Summary of case studies reporting olmesartan-induced enteropathy. F: female, M: male, N/R: not reported, AKI: acute kidney injury.

Study	Age/sex	Type of histopathology	Onset of symptoms	Symptoms	Outcome on discontinuation of olmesartan
Brandt et al. [5]	80/F	Ischemic colitis	Three weeks	Profuse diarrhea, weight loss	Improved
Ghaith and Szilagyi [7]	62/F	Intraepithelial lymphocytosis	N/R	Diarrhea	Improved
Emanuel et al. [8]	60/M	Villous atrophy, intraepithelial lymphocytosis	Two weeks	Watery diarrhea, AKI	Improved
Emanuel et al. [8]	87/M	Intraepithelial lymphocytosis	Two months	Watery diarrhea, weight loss, AKI	Improved
Shiho et al. [9]	73/M	Villous atrophy, collagenous colitis	Two months	Diarrhea, weight loss	Improved

The exact mechanism of olmesartan-induced enteropathy is not known. Angiotensin 1 (AT1) and angiotensin 2 (AT2) are the two types of angiotensin II receptors in humans. AT1 receptors are found throughout the alimentary tract and are essential in maintaining gut immune homeostasis by causing angiotensin II-mediated transforming growth factor-b signaling. AT2 receptors in the proximal portions of the small intestine are involved in the induction of intestinal epithelial cell apoptosis. Olmesartan has a greater affinity to inhibit AT1 receptors when compared to other ARBs. As the AT1 receptors are saturated by olmesartan, circulating angiotensin II may bind more likely to AT2 receptors, leading to pro-apoptotic effects and intestinal injury [4].

Intestinal ischemia is caused by a reduction in blood flow to an insufficient level to deliver oxygen and nutrients required for cellular metabolism [5]. With prolonged ischemia, irreversible damage with full-thickness ischemia can lead to transmural necrosis. Intestinal ischemia induced by drugs is commonly observed with constipation-inducing drugs, immunomodulator drugs, and illicit drugs like amphetamine and cocaine.

Bhattacharya et al. reported the first case of olmesartan-induced ischemic colitis in 2016 [6]. The patient presented with severe diarrhea, vomiting, abdominal pain, and weight loss, and the clinical symptoms improved with the discontinuation of olmesartan. In that case report, ischemia was in the colon, so a diagnosis could be made with a colonoscopy and biopsy. Our case is unique in that the ischemia happened in the jejunum and was complicated by perforation.

In our case report, the patient presented late after the onset of abdominal pain. This resulted in a delay in the discontinuation of olmesartan. We believe this could have contributed to the progression of the disease even after the discontinuation of olmesartan, resulting in bowel perforation. Moreover, the diagnosis was challenging owing to the location of the disease in the jejunum. The aim of our case report is to create awareness among physicians regarding the possibility of this grave complication. It allows them to better monitor for these symptoms while their patients are being treated with olmesartan.

With this case report, we recommend that there be a high level of suspicion for olmesartan-induced enteropathy, especially when patients have no significant comorbidities and present with unrelenting gastrointestinal symptoms while on this medication. In such situations, we also recommend that the physicians consider stopping olmesartan and starting on an alternative antihypertensive medication so as to prevent this complication.

## Conclusions

The aim of our case report is to increase awareness that there is a high chance of delayed diagnosis if we do not consider the possibility of olmesartan-induced ischemic enteritis. In our case, the patient presented five days after the onset of symptoms. Olmesartan was discontinued on the second day of admission as there was a high suspicion of drug-induced enteropathy. Delay in discontinuation of this medication after the onset of symptoms could be a predisposing factor that ultimately resulted in perforation of the bowel. Therefore, physicians prescribing this medication should be aware of the potential for enteropathies and may consider educating their patients to get medical advice immediately if they develop related symptoms. We also recommend more research be done on the mechanism of olmesartan-induced enteropathy.
